# Some critical methodological issues in secondary analysis of world health organization data on elderly suicide rates

**DOI:** 10.5249/jivr.v1i1.40

**Published:** 2009-07

**Authors:** Ajit Shah

**Affiliations:** ^a^International School for Communities, Rights and Inclusion, University of Central Lancashire, Preston, United Kingdom and West London Mental Health NHS Trust, London, United Kingdom,

## Abstract

**Background:**

Suicides may be misclassified as accidental deaths in countries with strict legal definitions of suicide, with cultural and religious factors leading to poor registration of suicide and stigma attached to suicide.

**Methods:**

The concordance between four different definitions of suicides was evaluated by examining the relationship between pure suicide and accidental death rates, gender differences, age-associated trends and potential distil risk and protective factors by conducting secondary analysis of the latest World Health Organisation data on elderly death rates. The four definitions of suicide were: (i) one-year pure suicides rates; one-year combined suicide rates (pure suicide rates combined with accidental death rates); (iii) five-year average pure suicide rates; and (iv) five-year average combined suicides rates (pure suicides rates combined with accidental death rates).

**Results:**

The predicted negative correlation between pure suicide and accidental death rates was not observed. Gender differences were similar for all four definitions of suicide. There was a highly significant concordance for the findings of age-associated trends between one-year pure and combined suicide rates, one-year and five-year average pure suicide rates, and five-year average pure and combined suicide rates. There was poor concordance between pure and combined suicide rates for both one-year and five-year average data for the 14 potential distil risk and protective factors, but this concordance between one-year and five-year average pure suicide rates was highly significant.

**Conclusions:**

The use of one-year pure suicide rates in cross-national ecological studies examining gender differences, age-associated trends and potential distil risk and protective factors is likely to be practical, pragmatic and resource-efficient.

## Introduction

Cross-national and single-country studies with an ecological design have conducted secondary analysis of data from the World Health Organization (WHO) on elderly suicide rates to examine age-associated trends,^1,2^ time trends ^2,3^ and potential distil protective and risk factors.^4,5^ However, findings from cross-national ecological studies should be viewed cautiously because: data are not available from all countries; ^6,7^ the validity of this data is unclear;^7,8^ the legal criteria for the proof of suicide vary between countries and in different regions within a country;^7,9^ some countries have poor death registration facilities;^9^ and, cultural and religious factors and stigma attached to suicide may lead to under-reporting of suicides.^7,10^ In countries with a strict legal definition of suicides, some possible suicides may be misclassified as accidental deaths. For example, in England and Wales, where the coroner can only return a verdict of suicide if suicide can be proved beyond a reasonable doubt, some genuine suicides may be misclassified as accidental death when suicide cannot be proved to this standard.^11-13^

Similarly, in countries with cultural and religious factors leading to poor registration of suicide and stigma attached to suicide, suicides may be misclassified as accidental deaths. If either or both of these scenarios are true then there would be a negative correlation between rates of pure suicide and accidental deaths. Although the vast majority of studies have used single year figures, several recent studies have used average suicide rates for five consecutive years to minimize the effect of year on year random fluctuation in suicide rates.^2-12^

Therefore, a study using the latest available data from the WHO was designed to examine: (i) the correlation between rates of pure suicides and accidental deaths; (ii) the concordance between age-associated trends in suicide rates using four different definitions of suicide; and (iii) the concordance for identified potential distil risk or protective factors using four different definitions of suicide. The four definitions of suicide were: (i) the one-year (the latest year) rate of pure suicides (ICD 9 code E54 or ICD-10 codes X60-84) – the one-year pure suicide; (ii) the one-year (the latest year) rate of pure suicide combined with one-year rate of accidental death (ICD10 X60-X84 combined with ICD-10 codes Y10-Y34) – the one-year combined suicide rate; (iii) the five-year (the latest five years) average rate of pure suicides (ICD 9 code E54 or ICD-10 codes X60-84) – the five-year average pure suicide rate; and (iv) the five-year (the latest five years) average rate of pure suicides combined with the five-year average rate of accidental deaths (ICD10 X60-X84 combined with ICD-10 codes Y10-Y34) – the five-year average combined suicide rate. Data on accidental death rates was not available for countries providing data on suicides rates using the ICD-9 code E54. The main underlying aim was to establish the best definition of suicide that could be used in future studies conducting secondary analysis of WHO data.

## Methods

The data on suicide rates and accidental deaths used in this study were the latest available and more recent than all previously published studies by the author’s group.

**1. Data on pure suicide rates**

Data on pure suicide rates (ICD-9 code E54 or ICD-10 codes X60-X84) for males and females in the seven agebands 15-24, 25-34, 35-44, 45-54, 55-64, 65-74 and 75+ years was ascertained from the WHO website (http:// www.who.int/whosis/database/mort/table1.cfm). For a small number of countries only the raw figures for the number of suicides were available (rather than suicide rates) from the WHO website. Pure suicide rates for these countries were calculated by dividing the number of reported suicides by the population size in the relevant age-band and sex group available on the same website. Data were ascertained for the latest five years. Data for the latest year were used for the one-year pure suicide rates. The median (range) for the year of the one-year pure suicide rate data was 2005 (1970-2007); the total number of countries with this data was 127.

The average pure suicide rate for the data for the latest five years was used to define the five-year average pure suicide rate. The median (range) for the latest year for the five-year average pure suicide rate data was 2005 (1983-2007); the total number of countries with this data was 97.

**2. Data on accidental death rate**

Data on accidental death rates (ICD-10 codes Y10-Y34) for males and females in the seven age-bands 15-24, 25-34, 35-44, 45-54, 55-64, 65-74 and 75+ years were ascertained from the WHO website (http:// www.who .int/whosis/database/mort/table1.cfm). For a small number of countries only the raw figures for the number of accidental deaths were available from the WHO website. Accidental death rates for these countries were calculated by dividing the number of reported accidental deaths by the population size in the relevant age-band and sex group available on the same website. Data were ascertained for the latest five years. Data for the latest year were used for the one-year accidental death rates. The median (range) for the year of the one-year accidental death rate data was 2005 (1999-2007); the total number of countries with this data was 91.

The average accidental death rate for the data for the latest five years was used to define the five-year average accidental death rate. The median (range) for the latest year of five-year accidental death rate data was 2005 (2000-2007); the total number of countries with this data was 60.

**3. Data on combined suicide rate**

Data for the latest year for pure suicide rates was combined with the data for the latest year for accidental death rates to form the one-year combined suicide rates (N=91). Data for the five-year average for the pure suicide rate was combined with the data for the five-year average for the accidental rate to form the five-year average for combined suicide rate (N=60).

**4. Correlation between pure suicide rates and accidental death rates**

Spearman’s correlation coefficient was used to examine the correlation between: (i) one-year pure suicide rates and one-year accidental death rates; and (ii) five-year average pure suicide rates and five-year average accidental death rates. These analyses were conducted for both sexes in the age-bands 65-74 and 75+ years.

**5. Gender differences**

The Wilcoxon’s matched-pair signed-ranked test was used to compare suicide rates between males and females for the age-bands 65-74 and 75+ years for each of the four definitions of suicide.

**6. Age-associated trends**

Each of the seven age-bands 16-24 years, 25-34 years, 35-44 years, 45-54 years, 55-64 years, 65-74 years and 75+ years were coded numerically in the ascending order of 1 to7. Spearman’s correlation coefficient (rho) was used to examine the relationship between the seven age-bands and the suicide rates by correlating the ascending order numerical codes for the seven age-bands with the suicide rate for each age-band. These analyses were conducted for both sexes for each country for each of the four definitions of suicide. This method of analysis has been successfully used to examine age-associated trends and time trends in elderly suicide rates.^1,2,14,15^ The actual data on the age-associated trends will not be described in this paper. However, the concordance for the findings of the age-associated suicide trends between (i) one-year pure suicide rate and five-year average pure suicide rate, (ii) one-year pure suicide rate and one-year combined suicide rate, and (iii) five-year average pure suicide rate and five-year combined suicide rate, was examined with the kappa coefficient.

**7. Potential risk and protective factors**

Studies conducting secondary analysis of data from the WHO and the United Nations Development Programme have reported associations between elderly suicide rates and gross national domestic product (GDP; a measure of socio-economic status),^4,16^ the gini coefficient (a measure of income inequality),^4^ life expectancy,^4^ child mortality rates,^4^ per capita healthcare expenditure,^4^ percentage of the GDP spent on health care,^4^ general population size,^4^ proportion of the population over the age of 60 years,^5^ fertility rates,^17^ the prevalence of smoking,^18^ educational attainment,^19^ Human Development Index ,^20^ degree of urbanisation,^21^ and population growth.^22^ Therefore, the association between suicide rates, for each of the four definitions, and GDP, the Gini coefficient, life expectancy, child mortality rates, per capita healthcare expenditure, percentage of GDP spent on health, general population size, proportion of the population over the age of 60 years, fertility rates and the prevalence of smoking was examined using Spearman’s correlation coefficient. Moreover, the curvilinear U-shaped relationship between suicide rates, for each of the four definitions, and the Education Index (a measure of educational attainment) and population growth following the quadratic equation (Y = A + BX + CX2, where A, B and C are constants, Y is the suicide rate and X is either the education index or population growth) was examined using curve regression estimates in accordance with previous literature.^19,22^ Furthermore, the curvilinear inverted U-shaped relationship between suicide rates, for each of the four definitions, and degree of urbanization and the Human Development Index following a quadratic equation (Y = A + BX - CX2, where A, B and C are constants, Y is the suicide rate and X is either the degree of urbanization or the Human Development Index) was examined using curve regression estimates in accordance with previous literature.^20,21^ The findings of the individual analysis for all the analyses listed in this paragraph will not be presented in this paper. However, the concordance for the findings for all fourteen variables examined between (i) one-year pure suicide rate and five-year average pure suicide rate, (ii) one-year pure suicide rate and one-year combined suicide rate, and (iii) five-year average pure suicide rate and five-year combined suicide rate was examined with the kappa coefficient.

 Data on GDP, per capita healthcare expenditure, percentage of GDP spent on health care, life expectancy, child mortality rates, general population size, degree of urbanization, percentage of population over the age of 65 years, population growth and fertility rates was ascertained from the WHO website (http://www.who.int/countries/afg/ en/) and were for the year 2006. Data on the percentage of population smoking (for years 2002-2004), the Gini coefficient (median year and range for the data was 2000 and 1992-2003), the Education Index (for 2004) and Human Development Index (for 2004) was ascertained from the United Nations Development Program website (http:// hdr.undp.org/en/media/HDI_2008_EN_Tables.pdf).

## Results

**1. Correlation between pure suicide rates and accidental death rates**

There was no significant correlation in males aged 65-74 and 75+ years between one-year pure suicide rates and one-year accidental death rates (N=91) and between five-year average pure suicide rates and five-year average accidental death rates (N=60). There were positive correlations in females aged 65-74 (rho=+0.46, P<00001; N=90) and 75+ (rho=+0.31, P=0.003; N=91) years between one-year pure suicide rates and one-year accidental death rates respectively. There was a positive correlation in females aged 65-74 years (rho=+0.39, P=0.002; N=60) between five-year average pure suicide rates and five-year average accidental death rates; this was not observed in females aged 75+ years (N=60).

**2. Gender differences**

As illustrated in Table 1 , male suicide rates were higher than female suicide rates in the age-bands 65-74 and 75+ years for all four definitions of suicide.

**Table:** Table 1: **Gender differences in suicide rates**

Pair	One-year pure suicide rate N=127	One-year combined suicide rate N=91	Five-year average pure suicide rate N=97	Five-year average combined suicide rate N=60
Males Vs Females 65-74 years	Z=-8.17, P<0.00001	Z=-7.5, P<0.0001	Z=7.79, P<0.00001	Z=-6.45, P<0.00001
Males Vs Females 75+ years	Z=-8.08, P<0.00001	Z=-7.12, P<0.00001	Z=-7.7, P<0.00001	Z=-6.09, P<0.00001

**3. Concordance for age-associated trends**

Table 2 illustrates the concordance between the findings of the age-associated trends analyses for both sexes between (i) one-year pure suicide rates and one-year combined suicide rates, (ii) one-year pure suicide rates and five-year average pure suicide rates, (iii) five-year average pure suicide rates and five-year average combined suicide rates, and (iv) one-year pure suicide rates and five-year average combined suicide rates. The kappa coefficient was 0.6 or higher and highly significant in all the analyses except when comparing one-year pure suicide rates with five-year combined suicide rates in females (0.53).

**Table:** Table 2: **Concordance for age-associated trends**

	Males	Females
One-year pure suicide rates Vs one-year combined suicide rates (N=91)	Kappa=0.74, P<0.00001	Kappa=0.6, P<0.00001
One-year pure suicide rates Vs five-year average pure suicide rates (N=97)	Kappa=0.7, P<0.00001	Kappa=0.73, P<0.00001
Five-year average pure suicide rates Vs five-year average combined suicide rate (N=60)	Kappa=0.72, P<0.00001	Kappa=0.65, P<0.00001
One-year pure suicide rates Vs five-year average combined suicide rate (N=60)	Kappa=0.66, P<0.00001	Kappa=0.53, P<0.00001

**4. Concordance for potential distil risk and protective factors**

Table 3 illustrates the concordance between the findings of the 14 potential distil risk and protective factors for both sexes in both the elderly age-bands between (i) one-year pure suicide rates and one-year combined suicide rates, (ii) one-year pure suicide rates and five-year average pure suicide rates, (iii) one-year pure suicide rates and those reported in the literature, (iv) one-year combined suicide rate and those reported in the literature, (v) five-year average pure suicide rates and five-year average combined suicide rates; (vi) five-year average pure suicide rate and those reported in the literature; and (vii) five-year combined suicide rate and those reported in the literature.

**Table:** Table 3: **Concordance for potential distil risk and protective factors**

	Males 65-74 Years	Males 75+ Years	Females 65-74 Years	Females 75+ Years
One-year pure suicide rates Vs one-year combined suicide rates	NS	NS	Kappa=0.26 P=0.018	NS
One-year pure suicide rates Vs five-year average pure suicide rates	Kappa=0.88 P<0.00001	Kappa=0.63 P=0.001	Kappa=0.74 P<0.00001	Kappa=0.65 P=0.001
One-year pure suicide rates Vs those reported in the literature	Kappa=0.53 P=0.005	Kappa=0.74 P<0.00001	Kappa=0.66 P<0.00001	Kappa=0.77 P<0.00001
One-year combined suicide rates Vs those reported in the literature	NS	NS	Kappa=0.45 P=0.04	NS
Five-year average pure suicide rates Vs five-year average combined suicide rate	NS	NS	NS	NS
Five-year average pure suicide rates Vs those reported in the literature	Kappa=0.65 P=0.001	Kappa=0.88 P<0.00001	Kappa=0.56 P=0.001	Kappa=0.78 P<0.00001
Five-year average combined suicide rates Vs those reported in the Literature	NS	NS	Kappa=0.35 P=0.01	NS

Median (range) number of countries for which data on different variables (N=14) was available: one-year pure suicide rates, 108 (46-108); one-year combined suicide rates, 74 (35-75); five-year average pure suicide rates, 85 (38-85); and five-year average combined suicide rate, 51 (29-52).

## Discussion

The “a priori” study hypothesis that there will be a negative correlation between pure suicide rates and accidental rates (because suicides may be misclassified as accidental deaths in countries with a strict legal definition of suicides and in countries with cultural and religious factors and stigma attached to suicide) was rejected because there was an absence of statistically significant negative correlation between (i) one-year pure suicide rates and one-year accidental death rates and (ii) five-year average pure suicide rates and five-year accidental death rates in both sexes in both the elderly age-bands. However, it is possible that this hypothesis may be true in individual countries which use strict legal definitions for suicide, with cultural and religious factors leading to poor registration of suicides and stigma attached to suicide. This hypothesis could be tested in such individual countries by comparing pure suicide rates and accidental death rates in longitudinal studies over time. The current findings suggest that the proposed inverse relationship between pure suicide rates and accidental death rates due to misclassification of suicides as accidental deaths may be less important in comparative cross-national studies.

Suicides rates were higher in males than in females in both the elderly age-bands for all four definitions of suicide and is consistent with a vast body of previous literature.^2,4^ The current findings suggest that any of the four definitions of suicide could be used to accurately examine gender differences in elderly suicide rates. Therefore, the use of one-year pure suicide rates in studies designed to examine gender differences in elderly suicide rates is likely to be practical, pragmatic and resource-efficient.

The concordance for the findings of age-associated trends in both sexes between (i) one-year pure suicide rates and one-year combined suicide rates, (ii) one-year pure suicide rates and five-year average pure suicide rates, and (iii) five-year average pure suicide rates and five-year average combined suicide rates was highly significant. Even the concordance for the findings of age-associated trends in both sexes between one-year pure suicide rates and five-year average combined suicide rates was highly significant. The current findings generally suggest that all four definitions of suicide provide similar results for age-associated trends in suicide rates in cross-national comparative studies. Therefore, the use of one-year pure suicide rates in studies designed to examine age-associated trends in suicide rates is likely to be practical, pragmatic and resource-efficient.

The concordance for the findings of the potential distil risk and protective factors in both sexes in both the elderly age-bands was generally poor and statistically not significant when comparing: (i) one-year pure suicide rates and one-year combined suicide rates, (ii) five-year average pure suicide rates and five-year average combined suicide rates; (iii) one-year combined suicide rates and the findings reported in the literature; and (iv) five-year average combined suicide rates and the findings reported in the literature. The lower number of countries with data on one-year and five-year average combined suicide rates may have resulted type 1 and type 2 statistical errors in detecting correlations between suicide rates and potential distil risk or protective factors. This, in turn, may explain the poor concordance between the findings for one-year and five-year average pure suicide rates and combined suicide rates. Moreover, the poor concordance between the findings for one-year and five-year average combined suicide rates and those reported in the literature may be explained by the observation that most previous studies used data on pure suicide rates.

The concordance for the findings of the potential distil risk and protective factors in both sexes in both the elderly age-bands was highly significant when comparing: (i) one-year pure suicide rates and five-year average pure suicide rates (kappa coefficient ranged between 0.63 and 0.88); (ii) one-year pure suicide rates and the findings reported in the literature (kappa coefficient ranged between 0.53 and 0.77); and (iii) five-year average pure suicide rates and the findings reported in the literature (kappa coefficient ranged between 0.56 and 0.88). Good concordance between the findings for one-year and five-year average pure suicide rates and those reported in the literature was not surprising because most previous studies, as described in the section on Methods, had used data on pure suicide rates. Nevertheless, this good concordance provides evidence of validity for the findings for one-year and five-year average pure suicide rates. Therefore, the current findings generally suggest that it may be more appropriate to use pure suicide rates in cross-national ecological studies examining potential distil risk and protective factors because: (i) the number of countries with available data on one-year and five-year average accidental death rates (and hence the combined suicide rates) is substantially lower and with the potential for type 1 and type 2 statistical errors; (ii) there was poor concordance for the findings for potential distil risk and protective factors between one-year and five-year average pure suicide rates and combined suicide rates; (iii) there was poor concordance for the findings for potential distil risk and protective factors between one-year and five-year average combined suicide rates and those reported in the literature; and (iv) there was an absence of negative correlations between pure suicide rates and accidental death rates. Moreover, the current findings suggest that it may be more appropriate to use one-year pure suicide rates in cross-national ecological studies examining potential distil risk and protective factors because there was good concordance for the findings of potential distil risk and protective factors between one-year and five-year average pure suicide rate. This is also likely to be practical, pragmatic and resource-efficient as only a fifth of the suicide rate data would require collection.

Overall, the current findings suggest that the use of one-year pure suicide rates in cross-national ecological studies examining gender differences, age-associated trends and potential distil risk and protective factors is likely to be practical, pragmatic and resource-efficient.
